# Lambda light chain-induced monoclonal gammopathy of renal significance, manifesting with Fanconi Syndrome and osteomalacia

**DOI:** 10.1186/s12882-022-02901-9

**Published:** 2022-08-09

**Authors:** Gabriel Brayan Gutiérrez-Peredo, José César Batista Oliveira Filho, Iris Montaño-Castellón, Andrea Jimena Gutiérrez-Peredo, Edvan de Queiroz Crusoé, Dimitri Gusmao-Flores

**Affiliations:** 1 University Hospital Professor Edgard Santos, Salvador, Bahia, CEP: 40110-060 Brazil; 2 Programa de Pós-Graduação em Medicina e Saúde, Universidade Federal da Bahia, Salvador, Brazil; 3 Gerência de Ensino, Pesquisa e Extensão - GEPE, University Hospital Professor Edgard Santos, Salvador, Brazil; 4 Nephrology Service, University Hospital Professor Edgard Santos, Salvador, Brazil; 5 Neurology Service, University Hospital Professor Edgard Santos, Salvador, Brazil; 6 Onco-Hematology Service, University Hospital Professor Edgard Santos, Salvador, Brazil

**Keywords:** Monoclonal Gammopathy of Renal Significance, Fanconi Syndrome, Type 2 tubular acidosis, Nephrology

## Abstract

**Background:**

Monoclonal gammopathy of renal significance (MGRS) encompasses a heterogeneous group of kidney diseases in which a monoclonal immunoglobulin secreted by a clone of B cells or plasma cells causes kidney damage without meeting the hematological criteria for malignancy. Among the various forms of involvement, MGRS can manifest as a proximal tubule disorder, such as Fanconi syndrome (FS), characterized by urinary loss of phosphate, glucose, amino acids, uric acid and bicarbonate. Few cases of MGRS have been described in the literature, manifesting as FS and monoclonal production of lambda light chains, almost all of which are secondary to the production of kappa light chains.

**Case presentation:**

Here we report a clinical case of a 45-year-old Brazilian male, African descent, with proximal weakness of the lower limbs, whose initial assessment showed a urine summary with the presence of proteinuria and glycosuria without hyperglycemia, associated with mild worsening of renal function, hypouricemia, hypocalcemia and phosphaturia. Evolution was characterized by a MGRS manifesting as FS and osteomalacia.

**Conclusion:**

The diagnosis of MGRS is not always easy, it requires knowledge of the clinical characteristics, diagnostic criteria and prognosis of each case. Therefore, all possible efforts should be made for multidisciplinary diagnosis.

## Background

Monoclonal gammopathy of renal significance (MGRS) is a disease that encompasses all kidney disorders caused by a monoclonal immunoglobulin (or fragment) secreted by a clone of non-malignant B cells, and that does not meet the criteria for hematologic malignancies such as symptomatic multiple myeloma or B-cell lymphoma [1, 2]. In the past, MGRS was part of the classification of nomoclonal gammopathy of undetermined significance (MGUS), representing approximately 10% of cases, with a prevalence ranging from 0.32% and 0.53% in patients over 50 and 70 years old, respectively [3].

Except in C3 glomerulopathy and thrombotic microangiopathy, renal lesions in MGRS occur due to abnormal deposition, regardless of serum concentrations, of immunoglobulins (IG)—whole or fractions of light, heavy or intact IG chains [4]. In addition, it can affect the glomerulus, tubule or vessels, generating a wide range of clinical manifestations, such as macroscopic hematuria, nephrotic syndrome, rapid loss of glomerular filtration rate, glucose metabolism and hydroelectrolytic/basic acid disorders—suggesting a proximal tubulopathy like Fanconi. MGRS can also be classified according to the characteristics of its deposits—organized or unorganized [3, 5].

The MGRS proximal tubule lesion by light chain has two morphological variants, with cytoplasmic inclusions and without inclusions [4, 6]. An uncommon complication of MGRS is Fanconi syndrome (FS), a proximal tubulopathy characterized by electrolyte abnormalities (phosphaturia, aminoaciduria, euglycemic glycosuria), metabolic acidosis and hypouricemia, typically positive for kappa light chain and less frequently for lambda chain in immunofluorescence [6–10]. It clinically presents with low back pain, bone pain, pathological fractures, fatigue or myalgia. Osteomalacia can be a complication associated with FS, resulting from hypophosphatemia, chronic metabolic acidosis and low levels of 1.25 hydroxy-vitamin D [7]. In general, light chain-associated Fanconi syndromes progress slowly to ESRD when untreated [11, 12], being associated with high morbidity [2] and histological recurrence after renal transplantation 30% [13].

The current therapeutic consensus suggests a clone-directed approach in case of MGRS-associated renal disease, i.e., antimyeloma agents in patients with plasma cell clones, rituximab-containing regimens in patients with lymphocytes or lymphoplasmacytic clone [5]. Treatment should also primarily aim to protect against the risk of progression to end-stage chronic kidney disease (ESRD), which brings high functional dependence, low quality of life and high risk of mortality [5, 14–16]. The prevention of thrombotic and infectious risk should be performed in cases of nephrotic syndrome, proteinuria and hypertension should be controlled, preferably with blockers of the renin-angiotensin system, and bicarbonate, phosphate and vitamin D supplements should be administered in patients with FS to prevent osteomalacia [5].

In MGRS patients with renal failure and an underlying plasma cell clone, regimens based on bortezomib, corticosteroids, cyclophosphamide and/or thalidomide can be used [17, 18], it is noteworthy that treatments with cyclophosphamide, high doses of melphalan, consolidated with autologous peripheral blood transplantation and autologous transplants of stem cells in some cases have also been successfully described [4, 5]. There are currently no randomized studies available for MGRS and treatment is mainly based on expert opinion or consensus; however, the ANDROMEDA multicenter clinical trial was recently published in patients with AL-amyloidosis, the trial studied Daratumumab, a human IgG-κ monoclonal antibody that targets CD38, this drug was associated with higher rates of complete hematologic response and survival free of major organ damage or hematologic progression, since AL-amyloidosis is a disease due to segregation of Monoclonal immunoglobulin, like MGRS, it could serve as a prototype example of a promising therapeutic alternative and reinforce the use of monoclonal antibodies in MGRS [19]. Renal transplantation in MGRS is controversial because recurrence has been observed, the decision of kidney transplantation must be taken into account the underlying characteristics of MGRS, initial therapy, patient's condition and the presence of extrarenal manifestations [5].

In this paper we present a clinical case of a previously healthy, middle-aged African descent man who sought medical care due to proximal paraparesis, and whose investigation determined a complete proximal tubulopathy (CPT) with an exuberant osteomalacia. Given the small number of similar cases reported, and the peculiarity of how it manifested as FS, lambda light chain and the of osteomalacia, our main objective is to share the clinical presentation, diagnosis and treatment of this rare disease, as well as its evolution after 2 years of clinical follow-up.

## Case presentation

A 45-year-old male patient reported weakness onset for two years in the lower limbs, progressing to the upper limbs and later tetraparesis, severe bone pain, especially at the lumbar level, and anserine gait, with significant functional limitation. He reported foamy urine, edema in the lower limbs and a recent fall from his own height due to muscle weakness – at that moment, already hindering his walking.

On physical examination, the patient was oriented, hemodynamically stable, normotensive, afebrile, normal cardiovascular examination and without visceromegaly. He reported pain in the costal arches, diffuse thoracolumbar pain that worsened with movement, breathing and walking. His extremities showed edema in the lower limbs (3 +) extending to the pretibial region, well perfused. Neurological examination revealed Gowers' sign, proximal tetraparesis, hypoactive osteotendinous reflexes in the lower limbs, preserved pale and arthrostatic sensitivity, anserine gait and right foot valgus.

The laboratory study revealed polycythemia in the blood count, in the biochemical alterations in renal function, hypokalemia, hypophosphatemia, hypouricemia and secondary acidemia, metabolic acidosis. These findings, added by the summary of urine with proteinuria (2 +) and glycosuria (2 + ; without dysglycemia), topographed a proximal tubulopathy. Serum calcium levels were normal but with an elevated alkaline phosphatase and a history of calcitriol replacement since admission to another institution (one year ago, calcitriol treatment was later suspended). The 1,25 dihydroxyvitamin-D was decreased and the viral serologies were negative. Proteinuria reached 4000 mg/24 h but always with normal serum albumin and renal ultrasonography showed no significant changes (Table 1.).

**Table 1:** Results of the laboratories

Variables	Reference values	At the other Hospital	Before chemotherapy	After chemotherapy
**Blood count**				
Red Cells	4.0–5.5 millions	6.3	5.4	5.3
Hemoglobin	12.5–17.5 g/dL	18.5	16.0	16.3
Hematocrit	40–50%	54.8	47.3	46.6
Leukocytes	4500–1000 mm3	9540	9270	15,800
Platelets	150,000–450,000/mm3	299,000	325,000	229,000
**Electrolytes**				
Phosphorous	2.5–4.5 mg/dL	1.4	2.0	3.2
Potassium	3.5–5.5 mEq/L	2.8	4.0	3.5
Calcium	8.8–11.0 mg/dL	9.3	9.0	9.1
Sodium	135–145 mmol/L	139	138	139
**Renal function**				
Creatinine	0.6–1.2 mg/dL	1.2	2.0	1.2
Urea	8–20 mg/dL	29	25	28
GFR (CKD/EPI)	≥ 90 mL/min/1.73m2	83	45	84
**Total proteins**	6.4–8.3 g/dL	6.9	7.0	7.5
Albumin	3.5–5 g/dL	4.3	4.6	4.4
Globulin	2–4 g/dL	2.6	2.5	3.1
**Urine Summary**				
Urinary pH	5–7	9.0	8.9	9.0
Density	1015–1025	1048	1024	1012
Protein	Absent	150 mg/dL	150 mg/dL	75 mg/dL
Glycosuria	Absent	300 mg/dL	300 mg/dL	100 mg/dL
Presence of crystals	Absent	Calcium oxalate	Amorphous phosphate	Urate
Cylinders				
**Proteinuria in 24/H**	< 150 mg/24H	2780	4000	636.6
**Gasometry**				
pH	7.35–7.45	7.2	7.2	7.3
PCO2	35–45 mmHg	40.2	45.1	29.7
pO2	80–100 mmHg	60.9	37.2	61.7
HCO3-	22–26 mEq/L	18.2	20.6	15.7
BE	-2/ + 2	-8.2	-4.2	-8.1
O2 saturation	95–97%	91	74.8	92.2
Ionic Calcium	1.1–1.3 mmol/L	1.3	1.1	1.2
FE-Potassium	10–20%	57.4	-	-
FE-Uric acid	2–10%	46%		
FE-Phosphorus	5%–15%	> 98.8	-	-
FE-Sodium	< 1% >	1.6	-	-
FE-Ureia	35–50%	67.6	-	-
FE-Creatinine	20–40%	46	-	-
Serum Osmolarity	285–295 mOsm/kg	294	-	-
**Others**				
Uric acid	3.4–7.0 mg/dL	2.1	-	-
PTH	14.0–72.0 pg/mL	23.9	-	15.7
1,25-OH-vitamin D	> 20	18.9	-	23.3
AF	40–129 U/L	969	322	233

*GFR* Glomerular Filtration Rate, *CKD/EPI* Chronic Kidney Disease Epidemiology Collaboration, *AF* Alkaline Phosphatase, *CPK* Creatine phosphokinase, *FE* Fractional Excretion, *H* hours, *PTH* Parathyroid hormone, *1,25-OH-vitamin D* 1,25-Hydroxy-vitamin D

**Fig. 1:**
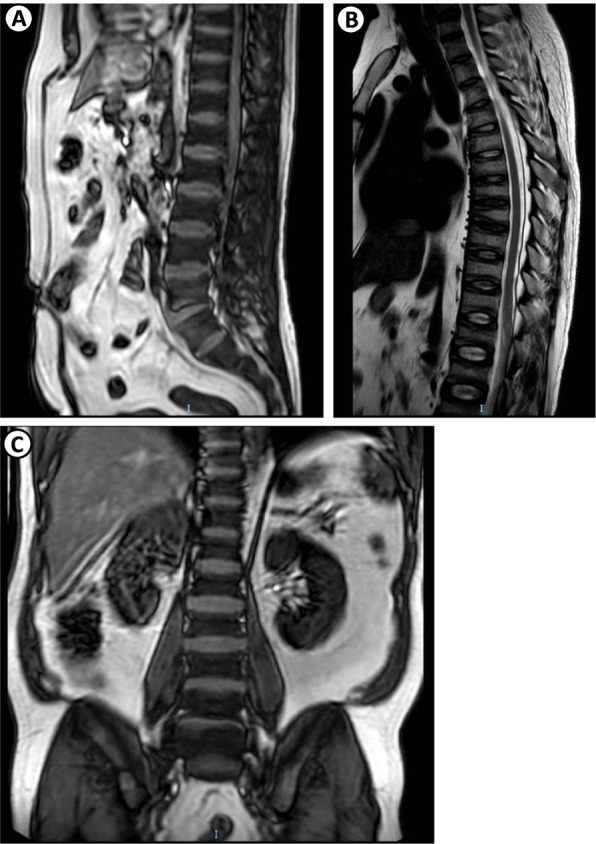
Magnetic Resonance. Vertebral bodies **A**, **B**, and **C** showing multiple, discrete height reductions on the upper and lower platforms, which are hyperdense sclerotic bands similar to the Rugger-Jersey pattern. This aspect may be related to systemic diseases of possible metabolism, and renal osteodysphrophy due to secondary hyperparathyroidism should be excluded. Image C. 0.3 cm microcalculus in the inferior calyx of the right kidney

Chest tomography showed misaligned fractures in multiple costal arches, related to a fall from one's recent height. Magnetic resonance imaging showed a Rugger-Jersey pattern (Fig. 1), X-rays of long bone, bone densitometry of the lumbar spine and femoral neck reported osteoporosis, compatible with osteomalacia.

In view of these findings, osteomalacia secondary to FS was suspected, and the search for the etiological diagnosis of FS was started. A 24-h immunofixation of urinary proteins was performed, which showed a monoclonal IgG lambda pattern, in the search for free light chains, a kappa of 14.4 mg/L (VR: 3.3–19.4 mg/L), lambda of 905.2 mg/L (VR: 5.7–26.3 mg/L) and kappa/lambda ratio of 0.02 (Table 2). Myelogram and bone marrow biopsy were also performed, with cellularity between 10–40% and less than 10% plasma cells. Hypoplastic erythroid and granulocytic series with maturation delay, small and hypolobulated megakaryocytes, unaltered reticulin pattern. Screening for amyloid protein was negative and it should be mentioned that bone marrow flow cytometry analysis was not performed.

**Table 2:** Urine protein immunoelectrophoresis, immunoglobulin immunofixation and free ligth chain research free

Variables	Reference values	Before chemotherapy	Afterchemotherapy
**Protein immunoelectrophoresis urine 24/H**			
Albumin	50–60% (8.0–29.9 mg/24H)	18.2%—592.0 mg/24H	25,3%—584.4 mg/24H
Alpha 1	4–7% (1.6–4.0 mg/24H)	7.3%—237.4 mg/24H	22,5%—519.7 mg/24H
Alpha 2	7–12% (3.2–6.4 mg/24H)	25.9%—842.5 mg/24H	30,80%—711.4 mg/24H
Beta	11–14% (4.8–7.2 mg/24H)	15.2%—494.4 mg/24H	11,8%—272.5 mg/24H
Gamma	14–21% (6.4–12.5 mg/24H)	33.4%—1086.0 mg/24H	9,60%—221.7 mg/24H
Total proteins	100% (24.0–60.0 mg/24H)	100.0%—3253.0 mg/24H	100%—2310 mg/24H
**Immunofixation of immunoglobulins (urine 24/H)**	-	Monoclonal IgG/Lambda standard	-
**Immunoglobulin free light chains**			
**(serum)**			
Kappa light chains	3.3–19.4 mg/L	14.4 mg/L	12.0 mg/L
Lambda ligth chains	5.7–26.3 mg/L	905.2 mg/L	32.2 mg/L
Relation kappa/lambda		0.02	0.37

*H* hours

To obtain values in g/L, divide the mg/dL by 100

To obtain values in g/24 h, divide the mg/24 h by 1000

**Fig. 2:**
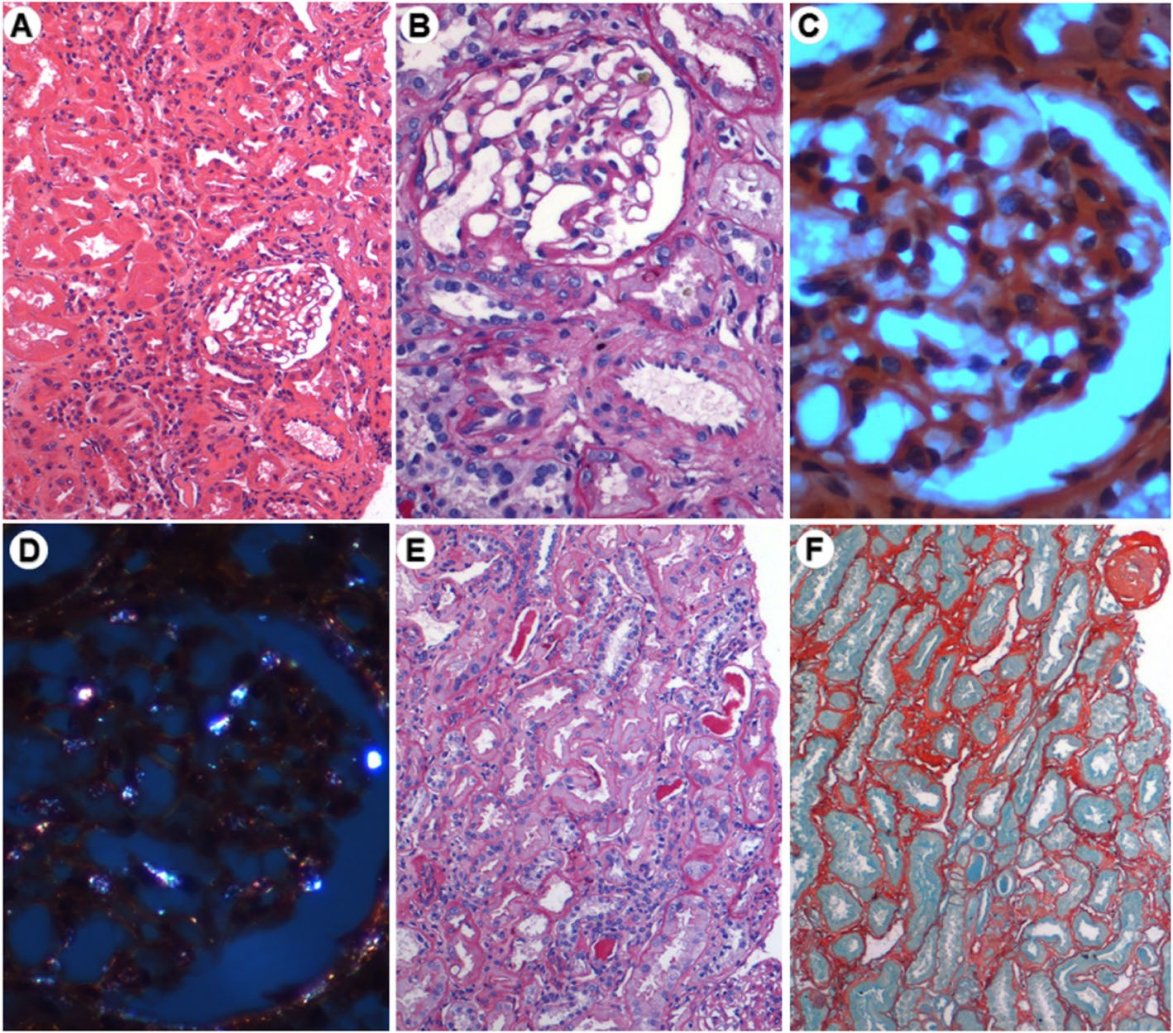
Renal biopsy—Optical microscopy, containing 3 glomeruli, 1 being totally sclerotic (**F**) and the other only partially represented. The remaining glomeruli unchanged (**A** and **B**). Congo Red stained sections (**C** and **D**) did not reveal amyloid material under polarized light (**D**). The tubules have epithelial regeneration foci, some hyaline casts (**E**) and atrophy foci (**F**—Picro Sirius). The interstitium had a mild focus of fibrosis (**F**). Unaltered arterial segments and arterioles. (A—H&E, B and E—PAS, C and D—Congo Red, F—Picro Sirius). Images courtesy of Dr. Washington LC dos-Santos, MD, IGM-FIOCRUZ

**Fig. 3:**
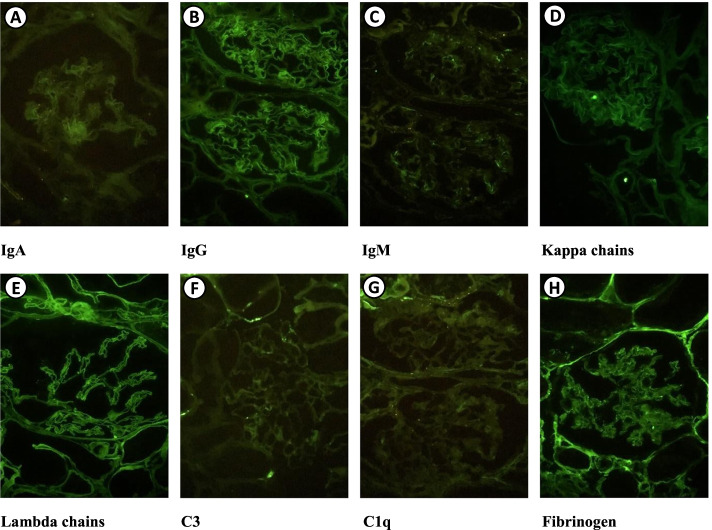
Renal biopsy—Immunofluorescence. Frozen renal biopsy sections, containing 4 glomeruli, incubated with a panel of fluorescent antibodies reveal: IgA (**A**), IgG (**B**), IgM (**C**), kappa chains (**D**), lambda chains (**E**), C3 (**F**), C1q (**G**) and fibrinogen all negative (**H**). Images courtesy of Dr. Washington LC dos-Santos, MD, IGM-FIOCRUZ

**Fig. 4:**
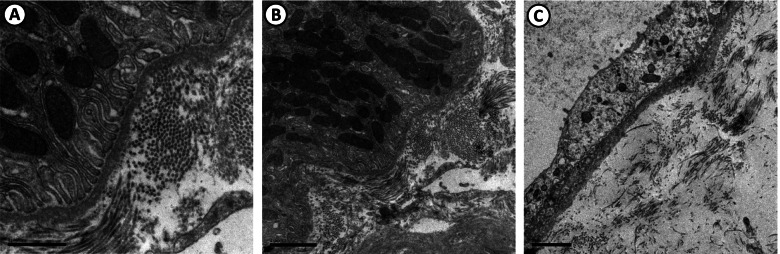
Renal biopsy—Electron microscopy. (**A**, **B** and **C**) Renal tubules with normal basement membranes or with mild thickening. There are no deposits of molecular aggregates. The tubular epithelium shows areas of thinning. Images courtesy of Dr. Washington LC dos-Santos, MD, IGM-FIOCRUZ

The renal biopsy showed only a fully sclerotic glomerulus on optical microscopy (Fig. 2), in the immunofluorescence the panel of fluorescein antibodies revealed negative for IgA, IgG, IgM, negative kappa chains, lambda, C3, C1q and fibrinogen chains (Fig. 3). In electron microscopy, no deposits were found in molecular aggregates and in the tubular epithelium, thinning areas were observed (Fig. 4). The Olimpus BX51 optical microscope (10X, 20X, 40X and 100X magnification lenses), the Spot-Flex camera for image capture and the Image-Pro Plus Software version 6.3 were used. The inverted optical fluorescence microscope and Normaski interferential contrast with imaging camera model DMi8, Leica and the transmission electron microscope JEM-1230 JEOL were used for immunofluorescence and electron microscopy.

In view of the above findings, in the initial approach, replacement was started with potassium citrate 10 mEq 8/8 h, calcitriol 0.25 mg 12/12 h, phosphorus 100 mg 12/12 h, powdered bicarbonate two teaspoons 12/12 h and enalapril 5 mg/day.

The proximal weakness led to ruling out the possibility of POEMS (Polyneuropathy, organomegaly, endocrinopathy, M-protein, skin changes), the electromyography showed no polyneuropathy, another laboratory was performed, the Vascular Endothelial Growth Factor (VEGF), 103.4 pg/mL (VR: < 128.9 pg /mL), which was negative, ruling out the possibility of POEMS.

Given the above, our team's understanding was not to submit the patient to a new biopsy, maintain support, measures for osteomalacia and treatment for monoclonal gammopathy of renal significance – whose hematology option was bortezomib 1.3 mg/m2 (2.28 mg) for 09 cycles, dexamethasone 20 mg weekly and oral cyclophosphamide 50 mg per day, with follow-up, monitoring of electrolytes, renal function, proteinuria and free-light chain.

With the start of treatment for MGRS and osteomalacia, the patient improved his clinical status, with a significant functional improvement self-reported by the patient himself (suspension of the use of crutches), corroborated by the laboratories that revealed a decrease in 24-h proteinuria, kappa/lambda ratio within normal range, eliminating the need for bicarbonate replacement and electrolyte replacement. Once considered as in remission of the disease, the treatment of monoclonal gammopathy was suspended.

## Discussion

Plasma cell and B cell dyscrasias generate an excess of immunoglobulin light chains, these can affect various organs. In general, the kidney can be affected in several segments: glomerulus, tubule, interstitium and vessel. In the kidney, up to 1 g/day of light chains are filtered and reabsorbed and catabolized by proximal tubular cells. When these light chains saturate the metabolic capacity of the proximal tubules, the unabsorbed light chains are detected in the urine as Bence Jones protein. Absorbed light chains can inhibit a variety of cellular functions, including organic ion transport, gluconeogenesis, and ammonia formation [6]. Light chain-induced nephropathy could manifest MGRS and cause proximal tubulapathy or Fanconi syndrome as in the present case, the manifestation will depend on the type and site of light chain deposition. In the case of our patient, the entry point was acid–base hydroelectrolytic disorders and non-nephrotic proteinuria, which, associated with glycosuria, promptly raised the possibility of FS. Over time, the patient developed Fanconi syndrome-induced osteomalacia secondary to MGRS.

Given the finding of the monoclonal peak, the presence of an altered K/L ratio, proteinuria and ruled out hematological disorders, nephrology ratified the MGRS impression and the need to start treatment. The need for renal biopsy in the context of Fanconi syndrome is somewhat controversial [2], but when we are facing a suspicion of MGRS it is mandatory [3, 14]. The association of Fanconi syndrome with lambda light chain proteinuria alone is rare, in the present study we report a case of symptomatic hypophosphatemic osteomalacia and Fanconi syndrome as a result of lambda light chain nephropathy and MGRS which makes it even rarer [6].

Faced with a renal biopsy sample (Image 2.) with only 3 glomeruli, 1 being totally sclerotic and 2 remaining unaltered, with immunofluorescence and electronics without glomeruli and with an unrepresented proximal tubule, we chose not to submit the patient to a new procedure and indicate the clinical treatment oriented by electrolyte disturbances, glomerular filtrate rate, proteinuria and K/L ratio.

The present case had no deposits in renal biopsy (Image 3.), but the monoclonal peak of urinary protein electrophoresis, such as immunofixation, was essential to guide the diagnosis, according to the International Kidney and Monoclonal Gammapathy Research Group (IKMG) renal biopsy was performed to maximize the possibility of a correct diagnosis [3]. We show the importance and rarity of this report of monoclonal MGRS lambda manifesting with FS and osteomalacia [20, 21]. Some patients may relapse and require autologous bone marrow transplantation, so the patient continued to undergo controls with nephrology and hematology. There was evidence of clinical and self-reported improvement by the patient, 3 months after starting treatment, with the consequent improvement in functional dependence and ambulation without the use of crutches.

## Conclusions

The diagnosis of monoconal gammopathy is not always easy, it requires knowledge of the clinical characteristics, diagnostic criteria and prognosis of each case. Therefore, every possible effort should be made for diagnosis. It is important to maintain knowledge of the MGRS in the practice of nephrology, neurology and hematology, maintaining a multidisciplinary work in the prevention of end-stage renal disease.

## Data Availability

The datasets used and/or analysed during the current study available from the corresponding author on reasonable request.

## Abbreviations

AF: Alkaline phosphatase
CKD/EPI: Chronic kidney disease epidemiology collaboration
CPK: Creatinophosphokinase
CPT: Complete proximal tubulopathy
ESRD: End-stage chronic kidney disease
FE: Fractional excretion
FS: Fanconi syndrome
GFR: Glomerular filtration rate
MGRS: Monoclonal gammopathy of renal significance
IKMG: International Kidney and Monoclonal Gammapathy Research Group
MGUS: Monoclonal gammopathy of undetermined significance
POEMS: Polyneuropathy, organomegaly, endocrinopathy, monoclonal protein, skin changes
RDW: Red cell distribution width
VEGF: Vascular endothelial growth factor
